# Biowaiver Monographs for Immediate-Release Solid Oral Dosage Forms: Meloxicam

**DOI:** 10.3390/molecules31061020

**Published:** 2026-03-18

**Authors:** Aixin Guan, Xueqiao Bei, Chan Jin, Jing Xie, Jianpeng Guo, Xiaoting Li

**Affiliations:** 1 Key Laboratory of Natural Medicines of the Changbai Mountain, Ministry of Education, College of Pharmacy, Yanbian University, Yanji 133002, China; guanaixin20@163.com (A.G.); guanaixin@foxmail.com (X.B.); 18059629571@163.com (J.X.); 2 Qilu Pharmaceutical (Hainan) Co., Ltd., Haikou 570311, China; jinchan0405@126.com; 3 Wuya College of Innovation, Shenyang Pharmaceutical University, Shenyang 110016, China; 4 Zhangzhou Vocational Institute of Technology, Health Science College, Zhangzhou 363000, China

**Keywords:** meloxicam, Bioequivalence Classification System, biological equivalence, biowaiver

## Abstract

This monograph evaluates the scientific and regulatory underpinnings for bioequivalence (BE) waiver of immediate-release (IR) solid oral meloxicam formulations, as a surrogate for in vivo pharmacokinetic trials. In compliance with ICH (The International Council for Harmonisation of Technical Requirements for Pharmaceuticals for Human Use), FDA (Food and Drug Administration), and PMDA (Pharmaceuticals and Medical Devices Agency) bioequivalence guidelines, a systematic characterization was performed on meloxicam’s critical attributes, encompassing solubility, permeability, dissolution behavior, pharmacokinetic profiles, therapeutic index, and drug-excipient compatibility. Classified as BCS Class II (low solubility, high permeability), meloxicam nonetheless exhibits a broad therapeutic window and pharmacokinetic characteristics aligning with BE Category I, thus enabling generic product approval via BE waiver with negligible risks of systemic exposure inequivalence. It is noteworthy that current in vitro methodologies are not consistently capable of capturing C_max_ disparities of BCS Class II weak acids. BE waiver eligibility for meloxicam IR formulations necessitates three prerequisites: (a) excipient composition identical to the reference listed drug and validated by regulatory authorities; (b) ≥85% dissolution within 30 min at pH 1.2, 4.5, and 6.8; (c) comparable dissolution profiles across these pH conditions. Non-adherence mandates a mandatory in vivo BE assessment.

## 1. Introduction

Meloxicam is a selective cyclooxygenase-2 (COX-2) inhibitor and one of the representative drugs among enolic acid-type nonsteroidal anti-inflammatory drugs (NSAIDs) [1]. As a BCS(biopharmaceutics classification system) Class II weak acid with a pKa of approximately 4.2, meloxicam exhibits pH-dependent solubility—poorly soluble at gastric pH (1.2–4.5) but highly soluble at intestinal pH (6.8–7.5)—while demonstrating high permeability and nearly complete oral absorption, with absolute bioavailability approaching 100% [2,3,4,5]. These biopharmaceutical characteristics, particularly the combination of high permeability and adequate solubility at the site of absorption, position meloxicam as a potential candidate for BCS-based biowaiver, provided that specific conditions regarding formulation and dissolution are met.

Given the widespread use of meloxicam in the long-term management of rheumatoid arthritis, osteoarthritis, and ankylosing spondylitis, and its inclusion in the WHO Model List of Essential Medicines [6], establishing a biowaiver pathway for its immediate-release, solid oral formulations has significant public health implications. Global projections indicate that rheumatoid arthritis, which affected approximately 17.6 million people in 2020, will rise to 31.7 million by 2050 [7,8]. These musculoskeletal disorders not only cause joint pain and functional impairment but also significantly diminish patients’ quality of life and increase healthcare burdens worldwide [9]. Reducing unnecessary in vivo bioequivalence studies can facilitate global access to quality-assured generic products while minimizing human exposure to clinical trials and reducing drug development costs.

This monograph systematically evaluates meloxicam against the regulatory framework for BCS-based biowaivers [10]. The recent WHO biowaiver guidance [11] and the ICH M9 guideline [12] establish the core criteria for eligibility, which are consistent with current expectations for bioequivalence assessment of immediate-release solid oral dosage forms [13]. According to these frameworks, biowaiver eligibility requires: (a) classification of the drug substance as BCS Class I or Class II with high permeability; (b) demonstration that the test and reference products are pharmaceutically equivalent; (c) evidence that excipients do not affect absorption in a manner that undermines bioequivalence; and (d) similarity of in vitro dissolution profiles across multiple pH conditions. The following sections examine meloxicam against each criterion to determine whether a biowaiver can be recommended for its immediate-release solid oral formulations.

Despite its low solubility at gastric pH, meloxicam meets the biowaiver eligibility criteria because absorption occurs primarily in the small intestine, where pH conditions (6.8–7.5) ensure rapid and complete dissolution. Clinical pharmacokinetic studies confirm meloxicam’s nearly complete oral absorption and high permeability [2,4,5]. Furthermore, biopharmaceutical research indicates that the current BCS solubility criteria may be appropriately interpreted for acidic drugs, as their ionization at intestinal pH does not preclude complete absorption [10]. Thus, for meloxicam, the key determinants of biowaiver suitability are not gastric solubility alone, but rather the combination of intestinal solubility, high permeability, and similarity of dissolution profiles between test and reference products.

The risk assessment for meloxicam biowaiver must consider formulation-specific factors that could affect its in vivo performance. First, dissolution profile similarity across multiple pH conditions (1.2, 4.5, and 6.8) is critical because pH-dependent solubility means that even minor formulation differences could alter dissolution at specific pH points, potentially affecting C_max_. Second, formulation processes and excipients require careful evaluation: certain excipients can dramatically increase meloxicam solubility in vitro, and while this may not always affect the extent of absorption (AUC), it could alter absorption rate (C_max_). Third, meloxicam’s pharmacokinetic features—long half-life (approximately 20 h), low interindividual variability, and complete absorption—provide a margin of safety, as minor formulation differences are less likely to push parameters outside standard bioequivalence limits (80–125%). Fourth, excipient interactions must be assessed to ensure that solubility enhancement does not lead to supratherapeutic exposure. These risk factors are particular to meloxicam because of its pH-dependent solubility, high permeability, and documented sensitivity to formulation variables.

This monograph systematically describes and evaluates the biopharmaceutical characteristics of meloxicam in accordance with the core principles of bioequivalence exemption based on biological equivalence as stipulated in the internationally recognized guideline (ICH M9), and explores the feasibility of granting a bioequivalence exemption (Biowaiver) for its immediate-release solid oral formulation [7]. The exemption methodology based on the Biopharmaceutical Classification System (BCS) includes: scientifically classifying the solubility and intestinal permeability of the active pharmaceutical ingredient (API); comprehensively comparing the in vitro dissolution profiles of the test formulation and the reference formulation in multiple pH media; and carefully evaluating relevant risk factors. These factors encompass in vitro dissolution curve similarity, formulation process effects on drug absorption, pharmacokinetic characteristics within the therapeutic dose range, and drug-excipient interactions.

Given meloxicam’s critical role in global disease management [10], applying BCS classification-based bioequivalence exemptions would significantly accelerate the development and market entry of high-quality generic versions, particularly in resource-constrained settings. The conclusions of this monograph provide a scientific rationale for regulatory authorities and the pharmaceutical industry to expedite access to this vital medication while ensuring therapeutic efficacy and safety, ultimately benefiting patients worldwide [11].

## 2. Discussion

### 2.1. Solubility

Reported experimental solubility data were determined by the shake-flask method and another justified method at 37 °C, in accordance with current BCS and biowaiver guidances. The highest dosage strength (according to the WHO, but this is not relevant for meloxicam as it is not on the EML) or the highest single dose administered (according to the EMA) guidance for “highly soluble” APIs ought to be soluble in 250 mL of aqueous solution at pH 1.0–6.8 (EMA) and 1.2–6.8 (WHO) while not adding surfactants. All guidelines suggest using at least three media to evaluate the solubility in the stated pH range [12,14]. Based on the aforementioned data, meloxicam exhibits low solubility under acidic pH conditions, while its solubility increases with rising pH. It becomes highly soluble at a pH greater than 5 (i.e., the pH of the duodenum) and remains highly soluble in simulated intestinal fluid as well [15,16]. The WHO BCS guidance permits a biowaiver to BCS Class II substances with weak acid properties and “high solubility” at pH 6.8. Thus, meloxicam(obtained from Egypt) would satisfy the WHO prerequisites for applying the biowaiver [17,18,19,20].

### 2.2. Absorption and Permeability

EMA and WHO permeability definition: For this purpose, complete absorption is taken into account to be established where the measured extent of absorption is ≥85%. Complete absorption is mostly associated with high permeability. The permeability criterion was relaxed from 90% in the FDA guidance to 85% in the WHO “Multisource document” [12]. Meloxicam is thought to be nearly completely absorbed after oral administration (FA about 89%), thus it is classified as a “highly permeable” compound [21]. In situ (intestinal perfusion in rats) and in vitro (Caco-2 cells) studies may support human in vivo data. Although log P can be largely related to human permeability, log P has not been recognized by regulators as a typical indicator of permeability [22]. The Biopharmaceutics Drug Disposition Classification System (BDDCS) classifies an API as “highly” permeable if its extent of metabolism exceeds 70% (or 90%) [23,24]. The extensive metabolism of meloxicam also suggests that it is “highly permeable”. In summary, meloxicam fulfills all common permeability criteria and can be clearly classified as a substance with high permeability [12].

### 2.3. BCS Classification

According to research data and relevant literature reports, meloxicam exhibits pH-dependent solubility, with lower solubility under acidic pH conditions and increased solubility as pH rises [18,25]. For acidic drugs, the solubility criteria may be overly stringent. A solubility limit at pH > 5 (the pH of the duodenum) may be more appropriate, as most compounds are primarily absorbed in the intestinal region. For ionizable compounds with high permeability under physiological conditions, considering an intermediate solubility classification appears meaningful; meloxicam is officially classified as a BCS Class II drug due to its pH-dependent solubility (poorly soluble at gastric pH). However, its high permeability and nearly complete absorption at intestinal pH mean that it exhibits characteristics resembling BCS Class I behavior under physiological conditions. This borderline profile supports the scientific rationale for considering a biowaiver for conventional immediate-release formulations of meloxicam, provided that rapid dissolution is demonstrated at pH 6.8–7.5. Importantly, this does not change its official BCS Class II classification. Regarding permeability, meloxicam demonstrates high intestinal mucosal permeability. Studies using the Caco-2 model have shown that its apparent permeability coefficient (Papp) is comparable to that of highly permeable reference drugs [23,24,25,26]. It is noteworthy that although it’s in vitro solubility at physiological pH is limited, under in vivo conditions, the drug forms micelles, and dissolves in the upper small intestine due to the influence of bile salts and lipids, thereby enhancing absorption. This mechanism may contribute to its relatively high oral bioavailability [27].

### 2.4. Surrogate Techniques for In Vivo Bioequivalence Testing

**Figure 1:**
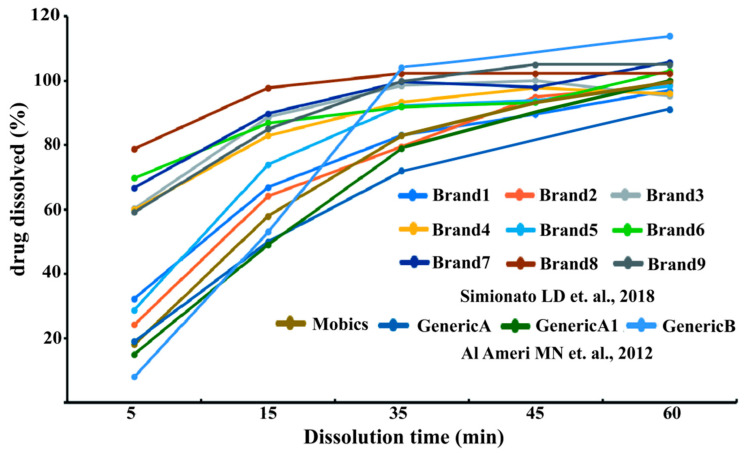
Examples of in vitro dissolution of different meloxicam tested medicines found in the literature using 900 mL of phosphate buffer pH = 7.5 in the USP Apparatus 2 apparatus at 75 rpm (Brand 1–Brand 9), or using 1000 mL of phosphate buffer pH = 7.5 in the USP Apparatus 2 apparatus at 50 rpm (Mobics, Generic A, Generic A1, Generic B). Data was digitized from Refs. [30,31], with Adobe Illustrator 2021.

The US FDA dissolution method database specify the dissolution method for meloxicam capsules: 500 mL (for 5 mg)/1000 mL (for 10 mg) of phosphate buffer at pH 6.8 with 0.1% SLS, using the basket I method at 100 rpm, with suggested sampling points at 5, 10, 15, 20 and 30 min; for meloxicam suspension: 900 mL of phosphate buffer at pH 7.5 using the Paddle II method at 25 rpm, with suggested sampling points at 5, 10, 15 and 30 min; and for meloxicam tablet: 900 mL of phosphate buffer at pH 7.5 using the Paddle II method at 75 rpm, with suggested sampling points at 10, 20, 30, 45 and 60 min [28]. Whereas both WHO and EMA indicate that the dissolution test of BCS biological exemption ought to be carried out in three dissolution media with pH values of 1.2, 4.5 and 6.8, at a rotational speed of 75 rpm in the paddle apparatus [12,14]. Ref. [29] conducted the same study at 50 rpm. The increase in rotational speed reduces the formation of soil piles. As shown in Figure 1, during the cone formation process, conducting dissolution studies on tablets at low rotational speeds (such as 50 rpm) may artificially affect the dissolution rate.

Anishetty et al. in their comparative study on the dissolution curve of the drug nanoparticles created into a solid oral dosage form and the marketed preparation (Muvera^®^), found that all meloxicam products tested did not meet the Pharmacopeia requirements for IR products in the tested PHS [29]. When a drug is transformed into a new drug delivery system, such as nanoparticles, it becomes more difficult to predict the behavior of the drug in vivo. The reason may be the presence of excipients used to make drug nanoparticles into solid oral dosage forms, which may change the disintegration and dissolution behavior of drugs, and may eventually lead to changes in bioavailability. At the same time, once considering formulations based on the solubility enhancement method, the comparative dissolution kinetics at low pH (especially pH 1.2) may be more discriminating. Specifically, the in vitro dissolution curve at pH 1.2 (rather than the in vitro dissolution curve at higher pH) seems to be appropriate for comparing drug products with or without solubilizers. Therefore, analysis at acidic pH may yield most of the variations, depending on the drug factor. However, as meloxicam is a weakly acidic drug, only a few bulk drugs are dissolved under acidic pH conditions, thus it is tough to distinguish the curve according to the *f*_2_ factor [30].

### 2.5. Risks with Respect to Excipient and Manufacturing Variations

The bioequivalence metric C_max_ may be influenced by various pharmaceutical factors, including the composition of excipients (such as fillers, surfactants, solubilizers, or agents that modulate gastric pH) and the particle size of the API. Surfactants such as meglumine, sodium bicarbonate, and sodium lauryl sulfate (SLS) significantly enhance the solubility of meloxicam. Patera et al. reported that co-grinding meloxicam with SLS (5–25% *w*/*w*) markedly increased the dissolution rate by approximately 100-fold. Moreover, the solubility of meloxicam increased linearly with rising meglumine concentration, reaching maximum values exceeding 28 mg/mL at 5 °C and 80 mg/mL at 25 °C—representing an approximately 10,000-fold improvement over its aqueous solubility [32]. Singh, S et al. demonstrated that a combination of sodium bicarbonate and cyclodextrin substantially improved both the solubility and dissolution rate of meloxicam, achieving over 90% drug release within 15 min in simulated gastric fluid [33]. Factors such as particle size, disintegration characteristics, supersaturation state, and disintegration time are known to significantly influence the in vitro performance of BCS Class II drugs. Extensive studies have confirmed that reducing the particle size of meloxicam, optimizing the disintegration characteristics of its formulation, employing specialized dosage form technologies to induce and maintain a supersaturated state, and shortening the disintegration time of meloxicam preparations can collectively enhance its dissolution rate and potential bioavailability [34,35,36,37,38].

Our research group previously conducted an in vitro comparison (according to the dissolution specification of meloxicam in the USP) and an in vivo bioequivalence study of two meloxicam preparations: rapid and slow dissolution. These preparations differ in many pharmaceutical factors, such as the content of lactose and poloxamer, all of which have an effect on the dissolution rate [18]. Each meloxicam preparation satisfies the dissolution specifications of the United States Pharmacopeia and is bioequivalent to the others. However, due to the slow disintegration time and dissolution rate of the “slow” meloxicam preparation in vivo, the C_max_ and AUC do not reflect the small difference in the dissolved dose fraction during gastric emptying or disintegration. These results highlight the trend of excessive discrimination in bioavailability results in vivo.

Simionato et al. conducted a dissolution study on commercially available drug products and found that there were significant differences in the dissolution curves of most products, although all of them met the requirements of the Pharmacopeia [30,31,39,40]. In contrast, all published studies on the bioequivalence of meloxicam reported the equivalence of the test product and the reference product in terms of AUC and C_max_. In addition, the dissolution curve test appears to have excessive discrimination against meloxicam drugs, and it is unlikely that the biological release test BCS will be rated as a false positive [18,29,41].

In brief, if a product contains only the excipients listed in this monograph and the content of these excipients in the test product is similar to that in the reference preparation, the risk of bioequivalence may be further reduced.

### 2.6. Patient’s Risks Associated with Bioinequivalence

The therapeutic index, treatment indications and side effects shall be evaluated when carrying out the biological exemption of API.

At vivlodex^®^ (by SoluMatrixTM technology) and Mobic^®^ in the comparison of dissolution and absorption, the results showed that the absorption of meloxicam in such preparations failed to rely upon excipients or manufacturing processes, indicating that the risk of bioequivalence in AUC was extremely low [18]. As previously discussed in the ketoprofen and piroxicam biowaiver monograph, the patient risk associated with the lack of bioequivalence of AUC is low. When taken together with food, the C_max_ of ketoprofen and piroxicam will be reduced by half; this phenomenon also applies to meloxicam (C_max_ decreases by about 20%) when taken with food [42]. Meloxicam is a long-acting NSAID primarily used for the treatment of chronic conditions such as osteoarthritis and rheumatoid arthritis, and its long half-life (approximately 20 h) means that for chronic therapy, the steady-state efficacy and safety are governed by the total systemic exposure (AUC) rather than the peak plasma concentration (C_max_) [43,44,45].

The patient risk associated with differences in C_max_ is necessary for NSAIDs used in the treatment of acute pain, because rapid pain relief is the main treatment goal. However, due to the low dissolution rate and slow action of meloxicam, meloxicam oral preparation is rarely used for the treatment of acute pain. It is mainly suitable for chronic treatment (persistent osteoarthritis, rheumatoid arthritis, and juvenile rheumatoid arthritis). Therefore, the risk of patients related to bioequivalence in C_max_ is smaller than that of patients suitable for acute treatment.

## 3. Methods

A comprehensive literature search was conducted using publicly available online databases, including PubMed, ScienceDirect, Google Scholar, and Scopus. The following keywords were used in various combinations: meloxicam, bioavailability, solubility, polymorphism, bioequivalence, pharmacokinetics, absorption, metabolism, excretion, pKa, distribution, BCS, excipients, biowaiver, and permeability. The search followed a narrative review approach rather than a formal systematic review with PRISMA-style screening, aiming to capture the breadth of available evidence relevant to the biopharmaceutical evaluation of meloxicam for biowaiver assessment. To ensure transparency and reproducibility, the following criteria were applied: (a) inclusion of peer-reviewed articles, regulatory guidance documents, and drug evaluation reports; (b) exclusion of non-English publications and conference abstracts without full data; (c) manual screening of titles and abstracts for relevance, followed by full-text review of potentially eligible articles. Regulatory sources included guidance documents and evaluation reports from the U.S. Food and Drug Administration (FDA), the European Medicines Agency (EMA), the Pharmaceuticals and Medical Devices Agency (PMDA) of Japan, and the International Council for Harmonization of Technical Requirements for Pharmaceuticals for Human Use (ICH) [12,13,14]. Relevant articles from journals specializing in pharmaceutical sciences and bioequivalence studies were also consulted. The last access date for all online literature was December 2025. In addition, partial data on meloxicam dissolution and release were derived from prior experimental evaluations conducted by our research group.

### 3.1. General Characteristics

**Figure 2:**
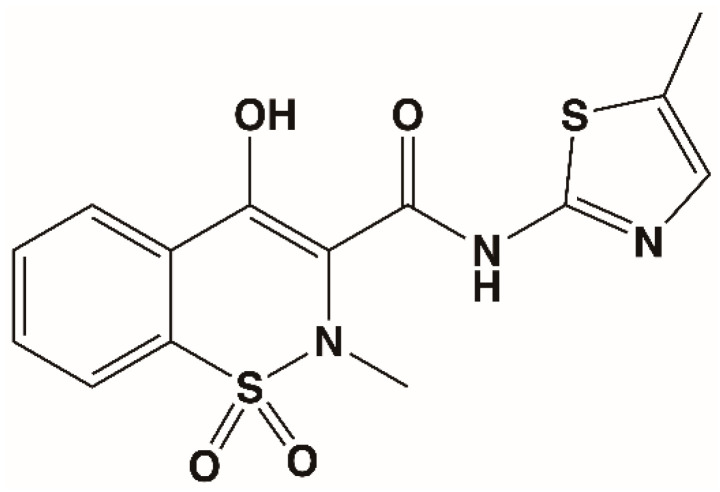
Meloxicam structure formula.

International Patent Name: Meloxicam [46,47]. Its chemical name is 4-hydroxy-2-methyl-N-(5-methyl-2-thiazolyl)-2H-1,2-benzothiazine-3-carboxamide 1,1-dioxide [47,48]. The chemical structural formula of this drug is shown in Figure 2. Meloxicam primarily exists as its sodium salt. It appears as a crystalline powder ranging from pale yellow to light yellow or pale yellowish-green to light yellowish-green, odorless. The molecular weight of meloxicam free acid is 351.4 g/mol, while that of the sodium salt is 391.4 g/mol. Therefore, 15 mg of meloxicam sodium salt is equivalent to 13.5 mg of meloxicam in its free acid form. Regarding polymorphism, meloxicam has been reported to exist in different polymorphic forms (e.g., Form I and Form II) under experimental conditions. As detailed in the comprehensive drug profile by Khalil and Aldosari [47], the thermodynamically stable form (Form I) is consistently used in pharmaceutical manufacturing, and form conversion during processing is well-controlled. Importantly, these polymorphic forms do not alter the fundamental BCS characteristics of meloxicam: solubility remains pH-dependent with poor solubility at acidic pH and high solubility above pH 5.0, and permeability remains high regardless of polymorphic form. Therefore, polymorphism does not clinically affect its solubility, dissolution, or BCS classification. Furthermore, drug-excipient compatibility studies by da Silveira et al. [48] have confirmed the solid-state stability of meloxicam in conventional formulations, indicating that no performance-altering polymorphic transitions occur under standard processing conditions. According to the ICH Q6A guideline [49], polymorphism testing is only required when different polymorphs have been shown to affect drug product performance; as no such issues have been reported for meloxicam, a detailed discussion was not considered essential in this section.

#### Therapeutic Indication, Dose, Therapeutic Index, and Toxicity

Meloxicam is a selective COX-2 inhibitor with half-maximal inhibitory concentrations (IC_50_) of 5.7 μM for COX-1 and 2.1 μM for COX-2 [1,50]. It belongs to the NSAIDs class of aliphatic acid derivatives. Its mechanism of action involves inhibiting cyclooxygenase (COX) to block the synthesis of inflammatory prostaglandins (PGs) [2,3]. Meloxicam is primarily indicated for treating chronic inflammatory conditions such as osteoarthritis and rheumatoid arthritis [21,47,51] while also demonstrating efficacy for acute pain, including postoperative or musculoskeletal pain [52]. Compared to traditional non-selective NSAIDs (e.g., ibuprofen or naproxen), meloxicam offers superior gastrointestinal tolerability at equivalent efficacy levels, making it suitable for patients intolerant to conventional NSAIDs. However, due to its COX-2 selectivity, caution is still warranted to mitigate cardiovascular risks [53]. Meloxicam dosages are typically expressed in tablet form. Clinical dosing requires individualized adjustment based on the patient’s condition type, age, hepatic and renal function status, and treatment response. Oral formulations are primarily available in 7.5 mg and 15 mg strengths (7.5 mg for elderly patients) [47,54]. In the United States, 7.5 mg and 15 mg meloxicam IR oral tablets, along with 5 mg and 10 mg meloxicam IR oral capsules [55], have received marketing authorization. Meloxicam IR formulations (tablets) are marketed in the European Union at doses of 7.5 mg and 15 mg. The dose strengths and therapeutic profile of meloxicam have direct implications for its biowaiver suitability. First, regarding dose strength, both the 7.5 mg and 15 mg doses fall well within the BCS solubility volume criteria, as documented in FDA-approved product information [4,55]. With a dose number (Do) calculated as (dose/250 mL)/solubility, the higher solubility of meloxicam at intestinal pH ensures complete dissolution of even the 15 mg dose within the 250 mL reference volume, supporting its eligibility for biowaiver consideration. Second, meloxicam possesses a relatively wide therapeutic index. Engelhardt demonstrated that meloxicam’s ulcerogenicity is weak in relation to its anti-inflammatory potency, resulting in a high therapeutic index [3]. Clinical studies have shown that doses up to 30 mg are well tolerated, and the drug exhibits low interindividual variability in pharmacokinetic parameters (CV ≈ 15–25%) [21]. Distel et al. further confirmed the favorable safety profile through a global analysis of clinical trials involving over 19,000 patients [51]. This wide therapeutic window provides a margin of safety in the context of biowaiver: minor formulation differences that might slightly alter C_max_ are unlikely to push plasma concentrations outside the therapeutic range or into toxic territory. Third, the safety profile of meloxicam informs the risk assessment for a biowaiver. Its superior gastrointestinal tolerability compared to non-selective NSAIDs reduces the consequences of any minor, clinically irrelevant formulation variations [3,51]. However, the recognized cardiovascular risks associated with COX-2 selective inhibitors—while low at therapeutic doses—warrant careful consideration, as noted in the FDA prescribing information [53]. Nevertheless, for immediate-release formulations that meet strict dissolution similarity criteria, the risk of clinically meaningful differences in exposure is minimal, and the established safety margin supports the appropriateness of a biowaiver for this drug. In the United States, meloxicam is approved for juvenile arthritis in children aged 2 years and older, with oral tablets indicated for pediatric patients weighing over 60 kg at a dose of 7.5 mg once daily [4,53]. The recommended oral dose is 0.125 mg/kg once daily (maximum dose 7.5 mg). Higher doses do not appear to confer additional benefit. In the UK, authorized product information does not approve meloxicam for adolescents under 15 years of age; however, the British National Formulary for Children (BNFC)—an authoritative prescribing guide for pediatric populations—provides off-label dosing recommendations for patients aged 12 to 18 years based on body weight: 7.5 mg daily for those weighing less than 50 kg, and 15 mg daily for those weighing 50 kg or more [4,39]. This inclusion reflects the clinical reality that meloxicam is used in pediatric practice despite regulatory label restrictions. These regional variations in pediatric dosing recommendations have implications for interpreting meloxicam’s therapeutic window and safety margin in the context of a biowaiver monograph. The fact that different weight-based dosing strategies exist—and that higher absolute doses (15 mg) are recommended for heavier adolescents in the UK despite the lack of formal approval—underscores the wide therapeutic index of meloxicam, which accommodates regional variations in dosing without compromising patient safety. For biowaiver assessment, the key consideration is not the specific approved dose in each region, but rather whether the dose strength under evaluation (typically 7.5 mg or 15 mg) falls within the BCS solubility volume criteria and whether the test and reference products demonstrate dissolution similarity. The established safety margin and favorable pharmacokinetic profile of meloxicam support the conclusion that minor regional differences in pediatric dosing recommendations do not fundamentally alter the risk evaluation for IR formulations. However, it is important to note that the BNFC recommendations represent off-label use; therefore, any biowaiver submission should reference the approved dosing in the target regulatory jurisdiction, while acknowledging that the wider therapeutic window supports the overall risk assessment [4,39].

**Figure 3:**
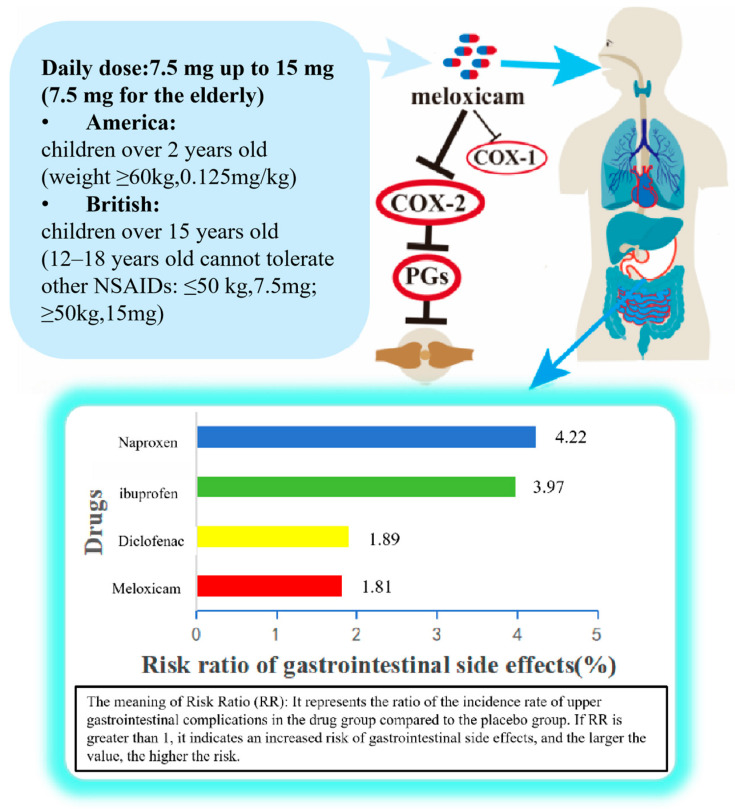
Meloxicam’s daily dose (suitable population in the United States and Britain) from Refs. [4,39,47], mechanism of action from Refs. [1,18] and the risk ratio of gastrointestinal side effects (data were digitized from Ref. [67]).

In multiple randomized clinical trials and long-term observations, meloxicam has been associated with gastrointestinal, cardiovascular, and renal toxicity, but no unique association with specific organ toxicity has been identified. The acute toxicity profile of meloxicam further supports its wide therapeutic window. The median lethal dose (LD_50_) values—84 mg/kg (oral, rat), 98 mg/kg (oral, female rat), and 800 mg/kg (oral, minipig)—indicate low acute toxicity across species [56]. These preclinical data, when considered alongside the clinical evidence of doses up to 30 mg being well tolerated in humans [21], reinforce the conclusion that meloxicam possesses a margin of safety sufficient to accommodate minor formulation differences that may arise in the context of a biowaiver. Compared to other NSAIDs, meloxicam exhibits dose-dependent characteristics in the incidence of gastrointestinal side effects and cardiovascular adverse events, as well as a risk of nephrotoxicity [57,58]. The incidence of non-serious adverse events is similar across NSAIDs: 43% in the 7.5 mg meloxicam (obtained from NY, USA) group, 45% in the 15 mg meloxicam group, 44% in the 20 mg piroxicam (obtained from NY, USA) group, 56% in the 100 mg diclofenac group, 61% in the 750–1000 mg naproxen (obtained from NY, USA) group, and 0.2% in the placebo group [50,59]. Recent studies indicate that meloxicam demonstrates comparable efficacy to naproxen, diclofenac, or ibuprofen in treating pain and inflammation associated with rheumatic and musculoskeletal disorders. At a daily dose of 15 mg, the risk of cardiovascular adverse events showed a significant difference only compared to piroxicam (*p* = 0.03), with a lower incidence; however, this observation may be limited to low-dose meloxicam [50,59,60,61]. The gastrointestinal safety profile of meloxicam has been extensively evaluated in large-scale clinical trials. The MELISSA study [62], a 12-week double-blind trial involving 9323 patients, demonstrated significantly fewer gastrointestinal adverse events with meloxicam (obtained from United Kingdom) 7.5 mg/day compared to diclofenac 100 mg/day (13% vs. 19%, *p* < 0.001), as well as fewer withdrawals due to gastrointestinal events (2.6% vs. 4.9%, *p* < 0.001). Similarly, the SELECT study [63], which enrolled 8656 patients over 26 weeks, reported a significantly lower incidence of perforations, ulcers, and bleeds with meloxicam (obtained from Belgium) 7.5 mg/day compared to piroxicam 20 mg/day (0.09% vs. 0.79%, *p* < 0.001). A global analysis of clinical trials by Distel et al. [51], involving over 19,000 patients, further confirmed this favorable safety profile [50]. As shown in Figure 3, the severe upper gastrointestinal event rate for meloxicam is approximately 1.81%, lower than that observed in diclofenac, naproxen, or ibuprofen groups [27,28]. Epidemiological studies have consistently demonstrated the reduced risk of upper gastrointestinal complications with preferential COX-2 inhibitors compared to traditional NSAIDs [57]. This superior gastrointestinal tolerability has direct implications for biowaiver risk assessment. First, it contributes to meloxicam’s wide therapeutic window: the margin between therapeutic and toxic doses is sufficiently large that minor formulation differences—which might slightly alter C_max_—are unlikely to result in clinically meaningful increases in gastrointestinal adverse events. Observational cohort studies have shown that meloxicam patients have significantly lower rates of reported gastrointestinal adverse drug reactions (1.80% vs. 3.20%) compared to other NSAIDs [60], and only 11% of patients discontinued therapy due to gastrointestinal adverse events in clinical trials [61]. Second, in the context of biowaiver, the key concern is whether formulation differences could lead to differences in patient outcomes. The established safety margin of meloxicam means that even if minor, clinically irrelevant variations in exposure occur, they are less likely to translate into meaningful differences in gastrointestinal tolerability. Therefore, the favorable gastrointestinal profile supports the overall benefit-risk assessment that underpins biowaiver consideration. It should be noted, however, that while the evidence for reduced upper gastrointestinal events is robust, a 2015 network meta-analysis comparing relatively selective COX-2 inhibitors with COXibs indicated that more data are needed to definitively confirm whether meloxicam has a lower incidence of intestinal side effects compared to other NSAIDs at current therapeutic doses [64]. Meloxicam is associated with an increased risk of cardiovascular adverse events, such as myocardial infarction, stroke, or thrombotic events, which may be potentially fatal. This risk may increase with prolonged duration of use. However, systematic reviews of cardiovascular risk with NSAIDs indicate that while COX-2 selective inhibitors warrant caution, the risk at therapeutic doses of meloxicam remains low, and the gastrointestinal benefits outweigh the risks for most patients [59]. Pharmacokinetic studies indicate that meloxicam exhibits stable plasma concentrations within the therapeutic dose range, with minimal interindividual variability [21,52,65]. While meloxicam is not classified as a narrow therapeutic index (NTI) drug [66], its therapeutic window is relatively broad. Individualized monitoring remains necessary to avoid the risk of overdose, particularly during long-term use or when co-administered with other medications.

### 3.2. Physicochemical Properties

#### 3.2.1. Stereoisomers, Salts, and Polymorphs

Meloxicam is known to have several pharmaceutical forms [68,69,70]. Coppi et al. predicted the existence of five crystalline forms of meloxicam called I, II, III, IV and V [68]. Unfortunately, the crystal structures of forms II and V are still unknown, and the crystalline form represented by IV may not be polycrystalline but a monohydrate solid [39]. Moreover, the Cambridge Structural Database (CSD) contains only one solvated structure of pure meloxicam (SEDZOQ and SEDZOQ01) [71]. Although the literature does not mention whether the polycrystalline form of meloxicam affects its bioavailability (BA), it is well known that polycrystalline forms lead to changes in the intrinsic dissolution rate and dissolution behavior of meloxicam formulations. Polymorphic form I is utilized in the manufacture of finished formulations of meloxicam. However, conversion of form I to form III may increase the solubility. At the same time, it may lead to a decrease in efficacy as form I is more active than form III [39,69,72,73,74].

#### 3.2.2. Solubility

According to published literature, pure meloxicam exhibits extremely low solubility [70]. It is described as practically insoluble in water in several pharmacopeias [71,72,75]. In the DrugBank database (Accession: DB00814), the aqueous solubility of meloxicam is listed as 7.15 mg/mL (25 °C), although the source and experimental conditions are not provided [76,77]. The solubility values determined by our group in preliminary experiments are summarized in Table 1. We observed that the solubility of meloxicam(obtained from China) increases with higher pH. At the 48 h time point, solubility was low at pH 1.0 and 4.5 (1.1 and 4.4 μg/mL, respectively). As a weak acid with a pKa of 4.1, the drug is highly soluble at pH 6.8 (0.235 mg/mL). At pH 1.0, 7.5 mg of the dose dissolves in 6637 mL; at pH 4.5, in 1685 mL; at pH 6.8, in 31 mL; and at pH 7.4, in 8 mL. Published solubility data for meloxicam in the presence of synthetic and natural surfactants indicate that incorporating sodium lauryl sulfate (SLS) and other relevant alternative surfactants into meloxicam solid dispersions does significantly enhance its solubility [15,16,78]. Dehghan et al. designed and prepared solid dispersions of meloxicam(obtained from India) in polyethylene glycol 6000 (PEG 6000) [26]. Solid dispersions containing PEG 6000 and the solubilizer SLS (with a meloxicam amount of 150 mg) showed a significant increase in dissolution rate with higher content of both PEG 6000 and SLS. Specifically, formulation 54 demonstrated the highest dissolution rate, with meloxicam release percentages of 82.69% at 30 min and 97.45% at 60 min. Shafique et al. employed spray-drying technology using surfactants and polymers to prepare unmodified crystalline meloxicam solid dispersions [79]. Using hydroxypropyl methylcellulose (HPMC) and SLS as hydrophilic carriers, various solid dispersions were prepared with different carrier/drug ratios. Among them, MSDs3 (with a meloxicam/HPMC/SLS ratio of 1:0.5:0.5) exhibited the highest solubility and dissolution (increasing meloxicam solubility from 0.25 ± 0.17 μg/mL to 153 ± 5.3 μg/mL). Furthermore, the dissolution at 15 min improved from 2.96 ± 0.55% to 47 ± 1.07%. Aejaz et al. found that ternary dispersions of meloxicam(obtained from South Korea) containing polyvinylpyrrolidone (PVP) and SLS, prepared via physical mixing and solvent evaporation strategies, showed a significant increase in dissolution rate with higher SLS content [78]. Sieger et al. observed that the solubility of meloxicam(obtained from India) increased approximately 10-fold in the presence of bile acids under both fasted and fed conditions (simulated using FaSSiF and FeSSiF media, respectively) [80]. Different crystalline forms of meloxicam demonstrate different solubilities. In a phosphate buffer at 37 °C and pH 7.5, the intrinsic dissolution rates (IDR) of meloxicam were 0.0672 mg/cm^2^·min for polymorph I and 0.0731 mg/cm^2^·min for a mixture of polymorphs I and III [57]. The D/S ratio was calculated based on the maximum single oral dose recommended in the prescribing information and the maximum single oral dose registered in ICH guidelines. Due to its poor aqueous solubility, various strategies have been employed to enhance the solubility of meloxicam, including particle size reduction, formulation modifications, complexation with β-cyclodextrin, the use of co-solvents, spray-drying technology, and co-crystallization [81,82,83,84,85,86,87].

#### 3.2.3. Partition Coefficient and pKa

The permeability classification of 123 oral drugs was based on the correlation between experimentally determined human intestinal permeability and the estimated log P, CLogP, or log D values of selected compounds. Metoprolol was chosen as the reference compound for permeability and log P, CLogP, or log D, since it is known that approximately 95% of this drug is absorbed from the intestine. Using metoprolol as the permeability reference, drugs with log P or CLogP values greater than or equal to the corresponding values of metoprolol (1.72 and 1.35, respectively) were classified as highly permeable [88]. Literature reports indicate that the partition coefficient of meloxicam ranges from −0.13 to 2.43 within a pH range of 2 to 12 [69]. Additional literature reports partition coefficients of 1.904 and 2.29 for meloxicam [81,89]. The pKa values for meloxicam are 1.09 and 4.18. The value of 1.09 is associated with the enolic OH group, whereas the value of 4.18 is associated with the nitrogen atom on the sulfathiazole ring [87]. According to the prediction software (MarvinSketch v.15.10.12.0 Software), the pKa values of pure meloxicam are 0.47 and 4.47. At the same time, the measured pKa values of pure meloxicam are 1.07 and 4.09 [90].

#### 3.2.4. Stability in Various pH Media

The stability of meloxicam under different pH conditions directly impacts the efficacy and safety of its formulations. Research data indicate that stability is poor in acidic environments (e.g., gastric fluid at pH 1.2), where degradation readily occurs. Stability improves under near-neutral pH conditions compared to acidic or alkaline environments. In alkaline conditions, stability decreases and degradation accelerates [91]. Conversely, the weakly alkaline environment (e.g., colonic fluid) facilitates colon-targeted release, making its stability suitable for formulation design [92].

### 3.3. Pharmacokinetic Properties

#### 3.3.1. Absorption and Bioavailability

Following oral administration, meloxicam is well absorbed from the gastrointestinal tract. Under fasting conditions, absorption is rapid and nearly complete after oral administration of conventional tablets or capsules. The absolute oral bioavailability of meloxicam(obtained from Germany) is 89%, with minimal first-pass effect, allowing the majority of the administered dose to enter systemic circulation and exert its effects [93]. The terminal elimination half-life of meloxicam is 13–24 h, with peak plasma concentration (C_max_) occurring 5–6 h post-dose [21,94]. A dedicated food effect study demonstrated that co-administration of meloxicam (obtained from Australia) tablets with a high-fat, high-calorie breakfast reduced the absorption rate, delaying T_max_ from 4.0 h in the fasting state to 10.0 h. However, total absorption was similar between fed and fasted states, with geometric mean ratios for C_max_ and AUC falling within bioequivalence criteria (80–125%) [94]. Pharmacokinetics of meloxicam are linear within the 7.5–30 mg dose range and remain consistent from single to multiple dosing. Steady-state plasma concentrations are achieved within 3–5 days. The observed stability of plasma concentrations following once-daily repeated dosing is attributed to meloxicam’s extended half-life.

#### 3.3.2. Permeability

No in vivo permeability data for meloxicam have been reported in the literature to date. Table 2 summarizes its permeability values obtained using different experimental methodologies. As an acidic drug, meloxicam exhibits enhanced permeability in acidic media, while a slight yet statistically significant reduction is observed under neutral pH conditions. According to the pH-partition theory, meloxicam permeability is pH-dependent: the unionized form predominates at low pH, favoring membrane permeation, while at intestinal pH, the ionized form predominates, which theoretically reduces permeability. However, despite this theoretical reduction, meloxicam demonstrates nearly complete absorption in vivo, supporting its classification as a high-permeability drug [26].

To predict bioavailability by simulating the gastrointestinal environment, a multi-pH gradient approach was employed for permeability measurement, with results correlated to findings from animal studies. This methodology introduces variability in permeability data for acidic and basic drugs across different pH levels [84]. In contrast, one study specifically investigated the effect of LogP on permeability under a fixed intestinal neutral pH, thereby reflecting permeability behavior only under that specific condition [94]. Another study shortened the cell culture period to 10 days to improve experimental efficiency; however, this abbreviated duration may compromise monolayer integrity and lead to deviations in permeability measurements [95,96]. Furthermore, research focusing on acidic drugs and associated dosages highlights the “discrepancy between regulatory standards and actual absorption” [25]. Collectively, these methodological differences contribute to the distinct patterns and variations observed in permeability data.

**Table 2:** Permeability data of meloxicam.

Method	Value	Acceptance Criterion	Permeability (Low/High)	Ref
Caco-2	P_app_AB = 54.7 × 10^−6^ cm/s			[26]
	P_caco2_ = 19.5 ± 6.2 × 10^−6^ cm/s P_accelerated_(AP → BL) = 39.5 × 10^−6^ cm/s			[94,96]
	P_(A to B)_ 7.5 mg = 17.6 ± 1.3 × 10^6^ cm/s	P_(A to B)_ > 1.57 ± 0.25 × 10^6^ cm/s	High	[25]
	P_(A to B)_ 15 mg = 13.8 ± 1.3 × 10^6^ cm/s			
	P_(B to A)_ 7.5 mg = 15.1 ± 0.6 × 10^6^ cm/s			
	P_(B to A)_ 15 mg = 15.3 ± 0.9 × 10^6^ cm/s			

P_caco2_: Caco2 cell permeability coefficient (cm/s). A: Apical side (the apical (luminal) side of the Caco-2 monolayer, which faces the intestinal lumen (or the donor chamber in a Transwell system)). B: Basolateral side (the basolateral (serosal) side, which corresponds to the blood circulation (or the receiver chamber)). P_accelerated_(AP → BL): an accelerated Caco-2 cell permeability model, permeability coefficient from apical to basolateral direction (AP → BL). All Caco-2 permeability studies were conducted at apical pH 6.5/basolateral pH 7.4, representing standard small intestinal conditions [95]. Although meloxicam (pKa ≈ 4.2) is predominantly ionized at this pH, the observed permeability values exceed the high-permeability threshold, consistent with its complete in vivo absorption documented in clinical studies [94]. This supports the view that acidic drugs can maintain high permeability despite ionization at intestinal pH [25].

#### 3.3.3. Distribution

Meloxicam is about 99.4% protein bound, primarily to serum albumin (>99%) [21]. Animal experiments show that meloxicam is especially distributed in high perfusion (albumin-rich) chambers such as blood, liver, and kidney [93,97]. This high protein binding leads to a restricted distribution volume (VD) of 10–15 L (about 0.1 to 0.2 L/kg), which is similar to other NSAIDs [21,98].

#### 3.3.4. Metabolism and Excretion

Meloxicam was nearly fully eliminated by metabolic degradation. The entire clearance rate of oral meloxicam was 0.42–0.48 L/h [98]. Meloxicam (obtained from Germany) experienced extensive first-stage reactions and no conjugated derivatives were detected. Most metabolites are formed by methyl oxidation of the thiazolyl moiety; further metabolites come from the cleavage of the thiazide ring system. Meloxicam metabolism is principally mediated by cytochrome P450, CYP 2C9 major pathway and CYP 3A4 minor pathway. Meloxicam is widely metabolized in the liver into four pharmacologically inactive metabolites (AF-UH 1, UH-AC 110 SE, BI-BO 8032 NA and DS-AC 2 SE), which are excreted through urine and feces. Schmid et al. gave four healthy male volunteers 30 mg ^14^C-labeled meloxicam as a short infusion and oral solution. During this study, trace (<0.25%) radiolabeled meloxicam was eliminated in urine, while just 1.6% of the parent compound was deposited in feces. This means that most metabolites of meloxicam were not associated with the route of administration. It is worth noting that there are significant changes in the distribution of metabolites in feces and urine. Urinary metabolites contain 18% AF-UH 1, 30% UH-AC 110 SE, 36% DS-AC 2 SE, and 11% BI-BO 8032 NA, while fecal metabolites contain up to 98% UH-AC 110 SE [99]. The excretion of drugs in the kidney and feces is roughly equivalent, with less than 0.25% of the parent compound contained in urine and 1.6% in feces. The latter may represent a small amount of unabsorbed drugs [21,93,97,99,100].

### 3.4. Dosage Form Performance

#### 3.4.1. Bioequivalence

No reports indicating a lack of bioequivalence of meloxicam IR formulations were found in the open literature. Much literature data has demonstrated the BE of meloxicam drug products. The details, BE criteria, and results of the BE studies are shown in Table 3. The studies showed that all tested products were bioequivalent to the reference products with respect to both AUC and C_max_.

**Table 3:** Results of published meloxicam bioequivalence studies.

Subjects (Females; Males)	Study Design	Drug Product (Manufacturer)	Dose (mg)	AUC (μg·h/mL)	C_max_ (μg/mL)	Criteria and Result	90% CI (AUC0–∞/AUC0–∞/Cₘₐₓ)	Ref
4 healthy volunteers (0:4)	single dose, two-period, crossover	Mobic^®^ (Boehringer Ingelheim, Singapore)	15	53.53	1.81	noncompartmental analysis, C_max_, AUC	AUC_0–∞_: 90.1–101.8%; C_max_: 87.5–99.3%	[17]
		EXT-SD (±SE)		77.81	2.43			
		FUS-SD (±SE)		41.87	1.33			
24 healthy volunteers (0:24)	open, randomized, two-period cross-over, single dose	(Melcam®, DEVA Holding A.Ş. Istanbul, Turkey; batch no. 4010178)	15	34.4990	1.1469	ANOVA, C_max_, AUC0–∞ 90% CI between 0.8 and 1.25 Bioequivalent	AUC_0–∞_: 90.1–105.3%; C_max_: 88.6–102.1%	[101]
		(batch no. 351558N)		33.7843	1.0648			
18 healthy volunteers (0:18)	open, single dose, crossover, two period	Melfax^®^ (AGP, Karachi, Pakistan)	15	28.367	1.023	*t*-test, C_max_, AUC Bioequivalent	AUC_0–t_: 91.5–101.2%; C_max_: 89.3–100.7%	[102]
		Xobix^®^ (Hilton Pharma, Karachi, Pakistan)		28.667	1.051			
24 healthy volunteers (15:9)	open-label, ran- domized, two-period, two-sequence, single dose, crossover	Reference: Meloxicam standard reference substance (batch 629/2453)	15	35.66701	1.30326	C_max_, t_max_, t_1/2_, AUD and AUC	/	[103]
		Test: Meloxicam 15 mg produced by LaborMed Pharma (Bucharest, Romania), batch 0903001462		37.31091	1.34541			
12 healthy volunteers (0:12)	single dose, randomized, two treatment, two periods crossover	Reference formulation (a local pharmaceutical company)	30	51.04 (AUC_0–t_)	1.48	ANOVA, C_max_, AUC_(0–t)_ 90% CIbetween 0.8 and 1.25 Bioequivalent	AUC_0–∞_: 92.8–104.6%; C_max_: 90.2–103.8%	[104]
		Test formulation (the innovator product)		47.12 (AUC_0–t_)	1.34			
24 healthy volunteers (9:15)	single-site, single-dose, randomized, open, 2-period, 2-sequence, crossover	Mobic	15	55.901 (Fasting)	1.854	C_max_, AUC 90% CI between 0.8 and 1.25 bioequivalence in both the fasting and fed states	Fasting AUC: 92.32–102.23%; Fed AUC: 91.98–102.01%; Fasting C_max_: 89.85–101.96%; Fed C_max_: 88.75–100.52%	[105]
				48.147 (Fed)	1.893			
		Aomei		59.042 (Fasting)	1.939			
				48.545 (Fed)	1.864			
4 healthy volunteers (0:4)	single dose, two-treatment, two period, randomized, crossover	conventional commercially available immediate release tablet (IR)	15	37.830	1.242	ANOVA, 90% CI between 0.8 and 1.25 Bioequivalent	AUC_0–t_: 89.6–101.5%; C_max_: 88.1–99.8%	[106]
		the selected optimum ODT formulation (F12)		40.189	1.589			
4 healthy volunteers (0:4)	9 healthy male Beagle dogs (10–15 kg), Single-dose, three-way crossover design	Mobic^®^(Boehringer Ingelheim)	7.5	117,074.89 ± 15,964.42	3194.86 ± 528.86	Bioequivalence Reference Criteria	/	[107]
		Meloxicam Tablets (Ningxia Kangya, Yinchuan, China)	7.5	116,343.80 ± 19,435.36	3136.15 ± 418.94	Meets bioequivalence	AUC0–∞: 90.5–108.5%; AUC0–∞: 90.1–110.1%; C_max_: 91.5–106.2%	
		HeChang^®^ Meloxicam Tablets (Ningxia Kangya)	7.5	114,092.57 ± 17,078.34	3081.30 ± 624.38	Meets bioequivalence	AUC0–∞: 88.9–106.6%; AUC0–∞: 90.4–110.5%; C_max_: 88.9–103.2%	

Abbreviations: EXT-SD, extended-release solid dispersion; FUS-SD, fused solid dispersion; ODT, orally disintegrating tablet; IR, immediate release; ANOVA, analysis of variance; CI, confidence interval; AUC, area under the curve; C_max_, maximum plasma concentration; t_max_, time to reach maximum concentration; t_1/2_, elimination half-life; AUD, area under the first moment curve; SE, standard error; Ref, reference.

#### 3.4.2. Effect of Excipients and Manufacturing Variations

According to the literature reports, the following excipients were used in the production of meloxicam tablets, nano-suspension solid formulations, and suspensions [107,108,109,110,111]. Tablets: Microcrystalline cellulose, sodium citrate, sodium carboxymethylcellulose, magnesium stearate, lactose, polyvinylpyrrolidone, corn starch, mannitol, and sodium bicarbonate. Water-based film coating ingredients include hydroxypropyl methylcellulose, titanium dioxide, glycerol triacetate, iron oxide red, and polyethylene glycol to enhance tablet stability and visual identification.

Nano-suspension solid composition ingredients: This formulation is produced by solidifying a nano-suspension. The core excipient is polyvinyl alcohol (PVA 0.5%) as the protective polymer. Carrier materials are divided into microcrystalline cellulose (for the fluidized bed process) and trehalose (for the freeze-drying process) based on the solidification method. No additional surfactants are added during production; dispersion stability is achieved solely through polymer adsorption. Suspension ingredients: Xanthan gum, sorbitol, methyl paraben, disodium EDTA, citric acid, sodium citrate, artificial strawberry flavor, and purified water. Some pediatric formulations add aspartame as a sweetener to improve medication compliance. Coating excipients, flavorings, colorants, flow aids, and lubricants are used in minimal quantities. Provided they meet pharmaceutical standards, they are generally considered unlikely to significantly affect the product’s critical quality attributes.

In summary, common excipients currently used in tablet and suspension manufacturing have been successfully applied to meloxicam oral formulations. Recent bioequivalence studies demonstrated that nano-suspension solid formulations produced via fluidized bed drying achieved nearly fivefold higher relative bioavailability compared to conventional nano-suspensions [111,112]. Additionally, excipient substitutions within regulatory limits (e.g., replacing part of mannitol with lactose) showed no significant impact on meloxicam’s in vivo absorption. For instance, Park et al. formulated meloxicam ODFs incorporating multiple excipients: polyethylene glycol 400 as a plasticizer, sodium hydroxide to enhance API dispersion by increasing meloxicam solubility, PVP K30 to prevent recrystallization of dispersed meloxicam molecules, and D-mannitol as a sweetener to improve patient compliance, whose physical properties also serve as an anti-caking agent [111].

#### 3.4.3. Dissolution

The USP39-NF34 dissolution specification for meloxicam tablets states that over 70% (Q + 5%) of the stated meloxicam amount should be released in 30 min in Apparatus II (paddle method) at 75 rpm in phosphate buffer (pH 7.5) at 37 °C [113]. Regulatory FDA dissolution conditions are phosphate buffer, pH 7.5, 900 mL, Apparatus II (Paddle) at 75 rpm, 37 °C [28]. The European Pharmacopoeia 7.0 does not include a monograph for the finished product, but provides a general dissolution standard: refer to Section 5.17.1, “Recommendations on Dissolution Testing”, for conventional release dosage forms, where not less than 75% (Q) of the labeled amount is specified to dissolve within 45 min [114].

There are several reports on the dissolution behavior of meloxicam IR formulations. Simionato et al. found that the nine brands of meloxicam tablets sold in Argentina all met the dissolution test specifications specified in USP 38 [30]. And the dissolution curve indicates that the analyzed product exhibits different dissolution curves, which may be related to the formula of the product (Figure 1). While Al Ameri et al. found that the in vitro dissolution of meloxicam brand products and generic drugs both met the pharmacopeia specifications and reached 85% dissolution within 60 min [31]. Anishetty et al. found that under different dissolution media (pH 1.8, 4.8 and 6.8), none of the tested meloxicam products met the requirements of the pharmacopeia for IR products. For IR formulations, many studies have shown that compendial in vitro dissolution tests with Apparatus II make it difficult to show the differences among them [29]. This is mainly related to the higher agitation level that causes the rapid disintegration of the tablet. Taha et al. reported that under varying experimental parameters utilizing a flow-through dissolution apparatus (USP Apparatus 4) with phosphate buffer (pH 7.5) as the medium, two out of the five multisource pharmaceutical products demonstrated comparable dissolution profiles under specified conditions. It was reported that the percentage of ionized meloxicam increased with increasing pH, with the highest solubility achieved at pH 10. On the other hand, the decrease in pH leads to an increase in the proportion of non-ionizing/ionizing drugs, thus leading to a decrease in solubility and dissolution [17]. Also, the results of meloxicam dissolution in all studied media clearly demonstrated the impact of manufacturing processes on the release behavior of meloxicam. Hence, a discriminatory dissolution procedure is a must requirement to differentiate the release behavior of a drug from a pharmaceutically equivalent product that contains different types and amounts of excipient in the formulation.

**Figure 4:**
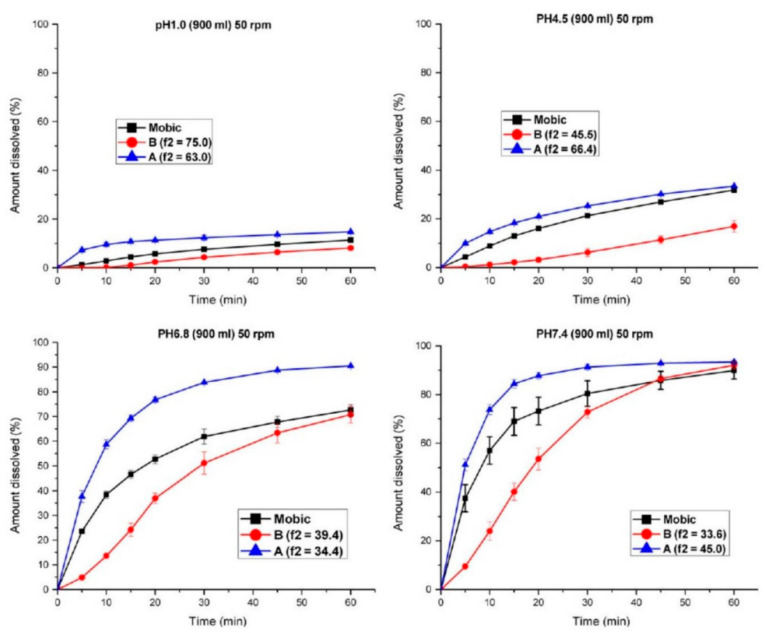
Comparison of dissolution profiles for product A or B versus the reference tablet (Mobica) in buffer media at pH values of 1.0, 4.5, 6.8, and 7.4 under a rotation speed of 50 rpm.

**Figure 5:**
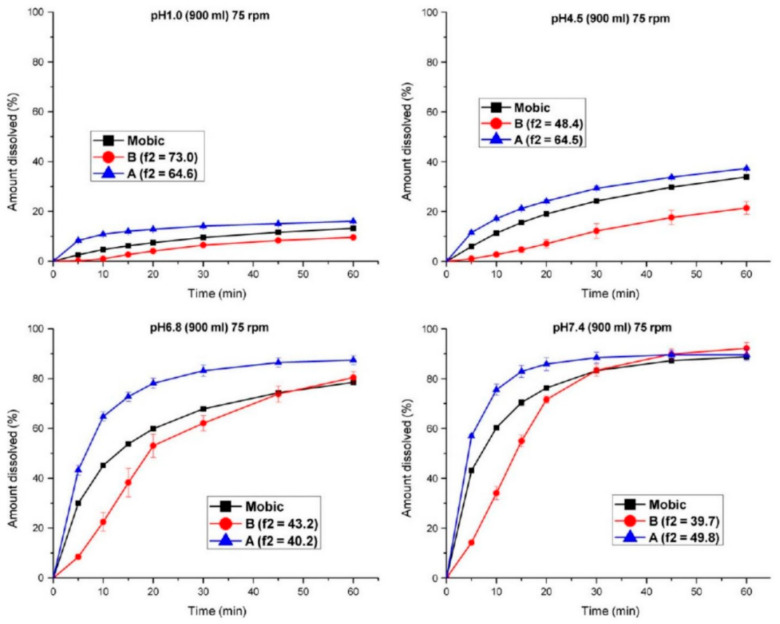
Comparison of dissolution profiles for product A or B versus the reference tablet (Mobica) in buffer media at pH values of 1.0, 4.5, 6.8, and 7.4 under a rotation speed of 75 rpm.

Meanwhile, investigations related to the medicinal product ought to ensure immediate-release properties and prove similarity between the investigative products, i.e., test and reference show similar in vitro dissolution under physiologically relevant experimental pH conditions. However, this does not establish an in vitro/in vivo correlation. In vitro dissolution ought to be investigated within the range of pH 1–6.8 (at least pH 1.2, 4.5, and 6.8) (Figure 4 and Figure 5). Additional investigations may be needed at pH values at which the drug substance has minimum solubility, and the use of any surfactant is not acceptable. Polymorphism represents a critical factor affecting drug solubility and dissolution performance [115]. Forms I and III are two common crystalline forms of meloxicam. Studies by Jacon Freitas et al. revealed that the API containing a mixture of forms I and III demonstrates approximately 1.8-fold higher solubility and about 1.5-fold greater intrinsic dissolution rate compared to pure form I, along with a corresponding 20–30% enhancement in tablet dissolution [39]. In contrast, form IV exhibits poor stability and is susceptible to dehydration. Research conducted by Antonio et al. indicated that during the dehydration process, the dissolution rate shows a slight decline during the transition from form IV to form V, followed by a marked reduction as form V converts to form I [116].

For BCS Class II weakly acidic drugs, there is often a lack of in vitro–in vivo correlation, where discrepancies may exist between in vivo bioequivalence results and in vitro dissolution outcomes, leading to certain misjudgments in dissolution evaluation [18,117]. In our previous study, a single oral dose of two formulations (7.5 mg) was administered to nine beagle dogs using a three-period crossover design, and in vitro dissolution tests were conducted in recommended media [117]. The results showed that the dissolution of meloxicam from the two test products differed significantly from that of the reference product in media with pH values of 1.0, 4.5, and 6.8 at agitation speeds of 50 or 75 rpm. However, the two test products were bioequivalent to the reference product in terms of both the extent and rate of oral absorption. Dissolution profiles intended to discriminate between formulations in vitro failed to accurately predict in vivo bioequivalence outcomes. Both test formulations remained comparable to the reference formulation in terms of C_max_. This can be attributed to the relatively long half-life (approximately 30 h) of the highly permeable meloxicam (obtained from MI, USA), where variations in in vivo dissolution may influence C_max_. Additionally, gastric emptying and tablet disintegration could be other significant factors affecting the absorption rate of meloxicam. However, the dissolution rate in the stomach is relatively low, making it slower than the gastric emptying rate, so most of the meloxicam likely remains in the stomach. Consequently, minor differences in the proportion of dissolved dose during gastric emptying or disintegration were not reflected in the C_max_ values.

To date, relevant studies have published data on the in vitro–in vivo correlation (IVIVC) of meloxicam extended-release solid oral dosage forms. Under fasting conditions, Taha et al. conducted single-dose (15 mg) and three-way crossover trials of MLX/SDS and Mobic^®^ in four healthy individuals. In vitro dissolution was also investigated in media with pH 1.2, distilled water (pH 6.4), and simulated gastric biorelevant media (pre- and postprandial) [17]. A Level A IVIVC was performed by comparing the fraction dissolved with the fraction absorbed over time, calculated using the Wagner–Nelson method. A multilayer Level C model for C_max_ and AUC0–96 was established and compared with the percentage dissolved at different time points. Both Level A and Level C IVIVC could predict the relationship between the entire in vitro dissolution profile (in distilled water and biorelevant FeSSGF) and the entire plasma drug concentration–time profile. Thus, without further in vivo studies, it was possible to establish and justify manufacturing site changes, minor formulation modifications, and scale-up attempts. These results highlight a tendency toward over-discrimination in in vivo bioavailability outcomes. Comparative dissolution profiles in media with pH values of 1.0, 4.5, and 6.8 appear overly conservative, which could lead to false-negative results. The comparative dissolution profiles using the similarity factor (f2) in recommended media should be relaxed to meet the requirements for the development, scale-up, and post-approval changes in meloxicam immediate-release oral solid dosage forms [18,118]. All of this evidence demonstrates that conventional pharmacopeial media cannot substitute for the in vivo performance of meloxicam (pKa 4.18) [29,119].

## 4. Conclusions

Meloxicam is a BCS Class II weak acid whose solubility increases with rising pH, exhibiting “high solubility” at elevated pH levels along with “high permeability.” For such an API, a biowaiver may be considered appropriate when the patient risk associated with bioequivalence concerning C_max_ is not critical and the risk of a false-positive biowaiver decision regarding AUC is minimal. Taking into account its BCS characteristics, the over-discriminatory tendency of dissolution testing for meloxicam formulations, as well as the clinical indications and experience with meloxicam, a biowaiver procedure can be recommended for immediate-release solid oral dosage forms containing meloxicam, provided the following conditions are met: (a) the test product contains only excipients that are identical to those in the approved IR solid oral drug product (containing meloxicam), and these excipients have been approved under ICH or relevant national guidelines, such as those referenced in this article; (b) if critical excipients are used, they should be of the same quality and in substantially similar quantities in both the test and reference products; (c) both the test and reference drug products demonstrate ≥85% dissolution within 30 min under pH conditions of 1.2, 4.5, and 6.8; and (d) the dissolution profiles of the test and reference products are similar at pH 1.2, 4.5, and 6.8. When any one or more of these conditions are not fulfilled, in vivo bioequivalence (BE) testing should be conducted.

## 5. Simple Summary

Meloxicam is a weak acid drug that dissolves better in higher pH environments. According to the Biopharmaceutics Classification System (BCS), it falls into Class II (low solubility, high permeability), meaning it dissolves slowly but is well absorbed once dissolved. This review examines whether immediate-release meloxicam tablets can qualify for a “biowaiver”—meaning they can be approved based on laboratory dissolution tests instead of expensive human studies. We conclude that a biowaiver is appropriate for meloxicam, but only under strict conditions. First, the generic product must contain the same inactive ingredients (excipients) as the reference product, in similar quantities. Second, both products must dissolve rapidly (≥85% within 30 min) under three different pH conditions (1.2, 4.5, and 6.8). Third, their dissolution patterns must be similar across these pH levels. If any of these conditions are not met, human bioequivalence studies are still required. This approach balances patient safety with regulatory efficiency, ensuring that safe and effective generic meloxicam reaches patients while avoiding unnecessary testing.

## Figures and Tables

**Table 1:** Number of doses based on meloxicam solubility.

Acidity (pH)	Dose Strength (mg)	Solubility C_s_ (mg/mL)	D_0_
1.0	7.5	1.139 × 10^−3^	26.55
4.5	7.5	4.459 × 10^−3^	6.74
6.8	7.5	0.24	0.13
7.4	7.5	0.95	0.03

D_0_ = M/V/C_s_, where “M” is the dose of meloxicam (7.5 mg), “V” is the initial gastric volume (250 mL), and “C_s_” is the solubility of meloxicam.

## Data Availability

All data supporting the findings of this review are derived from publicly available sources and have been cited accordingly. The original contributions presented in the study are included in the article; further inquiries can be directed to the corresponding author.
